# The anti-cancer properties of heparin and its derivatives: a review and prospect

**DOI:** 10.1080/19336918.2020.1767489

**Published:** 2020-06-14

**Authors:** Sai-Nan Ma, Zhi-Xiang Mao, Yang Wu, Ming-Xing Liang, Dan-Dan Wang, Xiu Chen, Ping-an Chang, Wei Zhang, Jin-Hai Tang

**Affiliations:** a Department of General Surgery, The First Affiliated Hospital of Nanjing Medical University, Nanjing, P.R. China; b Department of Oncology, The Affiliated Suqian Hospital of Xuzhou Medical University, Suqian, P.R.China; c Department of Oncology, Affiliated Hospital of Xuzhou Medical University, Xuzhou, P.R. China; d Core Facility, The First Afﬁliated Hospital of Nanjing Medical University, Nanjing, P.R. China; e Urinary Surgery, Dongtai People’s Hospital, Dongtai, P.R. China

**Keywords:** Heparin, low-molecular-weight heparin, heparin derivatives, cancer, metastasis

## Abstract

Heparin, including unfractionated heparin (UFH), low-molecular-weight heparin (LMWH) and heparin derivatives, are commonly used in venous thromboembolism treatment and reportedly have beneficial effects on cancer survival. Heparin can affect the proliferation, adhesion, angiogenesis, migration and invasion of cancer cells via multiple mechanisms. The main mechanisms involve inhibition of heparanase, P-/L-selectin, angiogenesis, and interference with the CXCL12-CXCR4 axis. Here we summarize the current experimental evidence regarding the anti-cancer role of heparin and its derivatives, and conclude that there is evidence to support heparin’s role in inhibiting cancer progression, making it a promising anti-cancer agent.

## Introduction

While humans enjoy an unprecedented level of technological advancement that supports increasing lifespan, researchers continue to struggle to find treatments to combat the rising incidence of cancers. Lung cancer is the leading cause of cancer death in men worldwide and has surpassed breast cancer to be the leading cause of cancer death in women in developed countries. However, the mortality rate of breast cancer is still the highest among all cancers in women of less developed countries. In addition, colorectal cancer, liver cancer, stomach cancer, cervical cancer, pancreatic cancer, and prostate cancer all present major worldwide threat to human health [1]. Conventional cancer treatments including surgical resection, chemotherapy, target therapy, radiation therapy, and immunotherapy, have all achieved positive results. Nevertheless, these treatments are not effective for a substantial number of patients with advanced or drug-resistant cancer, and there is a pressing need to develop alternative treatments. A novel potential has arisen from the coincidental need to treat cancer patients for blood hypercoagulability. Patients with advanced cancer including multiple metastases are often required to spend long stretches of time in bed, significantly increasing the risk of venous thromboembolism. Heparin, a polydisperse mixture of glycosaminoglycans (GAGs) has strong anticoagulant effects, and along with its derivatives is widely used in anticoagulation treatment to prevent venous thromboembolism. Serendipitously, when treating at-risk cancer patients, heparin and related drugs have been found to have anti-cancer functions. In this article we review the current evidence that heparin and its derivatives have anti-cancer properties, and we highlight both the potential for heparin in cancer treatments, and the challenges to its successful application.

## Chemical characteristics of heparin and its derivatives

**Figure 1.**
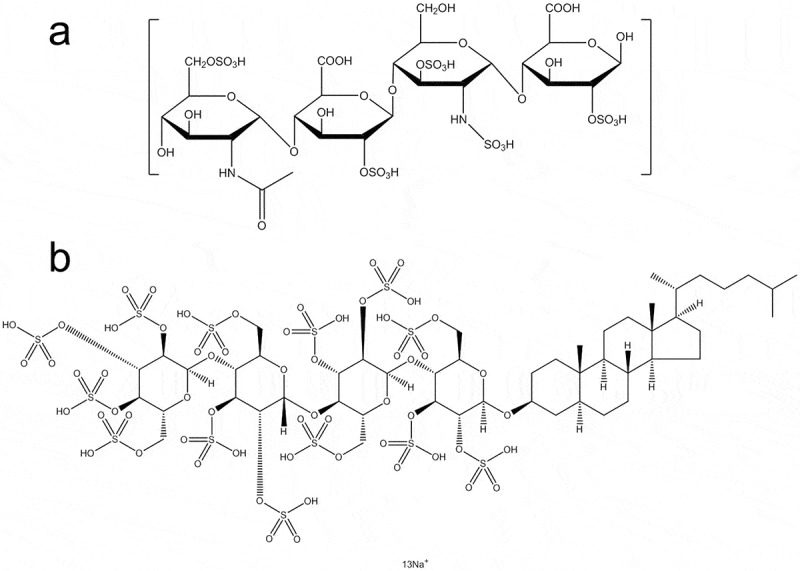
Molecular structure of heparin and its derivatives. (a) A representative monomeric chemical structure of glycosaminoglycan (GAG) and LMWH. (b) Chemical structure of PG545.

Heparin is a complex mixture of natural GAG isolated from porcine intestine and is usually prepared as a sodium salt. Heparin is classified into two types, unfractionated heparin (UFH) and low-molecular-weight heparin (LMWH), with the latter type including a number of subtypes such as enoxaparin, nadroparin calcium, dalteparin sodium, and tinzaparin. With the development of modern biosynthesis methods, many new types of heparin have been synthetically modified by adding or replacing some heparin chemical groups (Figure 1)[2]. Summary of all abbreviations used in this review is presented in Table 1.

Heparin derivatives include heparin-like glycosaminoglycans (HLGAGs), sulfated non-anticoagulant heparin (S-NACH), low-molecular-weight heparin-taurocholate-tetramer deoxycholate (LHTD4), LHTD4/DCK (a complex of LHTD4 and deoxycholylethylamine DCK), high-molecular-weight Escherichia coli K5-derived heparin-like polysaccharide (K5-NSOS), LHsura (a complex of heparin and suramin fragment), and LHbisD4 (a conjugation of low molecular weight heparin and four bis-deoxycholates). Heparin binds with a wide range of proteins, so that it has a diverse ability to regulate protein functions.

## Anti-cancer ability of heparin and its derivatives

While heparin and its derivatives can benefit cancer patients as anticoagulants, they directly impact on cancer progression via anti-metastatic effects [3–5]. Compared with UFH, LMWH can improve the 3-month and 6-month survival of cancer patients [6–11], and HLGAGs reportedly have a similar effect [12,13]. Initially it was thought that heparin’s anti-metastatic effects were via antithrombotic mechanisms, but more recent research suggests that the anti-cancer effect reflects an independent property [14–18]. A multicenter clinical trial exploring the influence of anticoagulant treatment in 277 small cell lung cancer patients showed that 5-week subcutaneous heparin treatment led to substantially improved survival rates compared to no treatment at 1, 2 and 3 years (40% vs. 30%, 11% vs. 9%, and 9% vs. 6%, respectively) [19]. Another clinical study found that death rates in ovarian cancer patients at a 2-year postoperative follow-up were 24% following treatment with certoparin compared to 37.5% following treatment with unfractionated heparin (UFH), suggesting that LMWHs are better than unfractionated heparin (UFN) in improving survival rates [20]. Additional studies have found evidence that heparin and its derivatives reduce the emergence of metastatic lesions and prolong survival in cancer patients.

Taken together, extant studies suggest that heparin and its derivatives confer a survival benefit in cancer, and optimizing the potential for effective treatment requires understanding the underlying mechanisms. A range of studies suggest heparins suppress tumor growth and metastasis by inhibiting tumor growth factors or angiogenesis, suppressing lymphatic vessel formation, reversing multidrug resistance, generating heparinase and thrombin, or inhibiting adherence of cancer cells to vascular endothelium [21–23]. Moreover, different heparin derivatives target specific biological mechanisms to inhibit tumors (Table 2).

**Table 2.** Heparin and its derivatives in different tumors and related mechanisms.

Cancer types	Study model	Type	Beneficial effects	Target molecular	Total Numbers of References
Breast	HDLECs, 4T1 cells, MDA-MB-231 cells	LHbisD4	Decreasing lymphatic vessels and attenuating lymph node metastasis	VEGF-C/VEGFR-3	27
	C_3_H mice breast cancer model	LMWH	Inhibiting lung metastasis	VEGF	31
	MDA-MB-231 cells	tinzaparin	Inhibiting pulmonary metastasis	CXCL12-CXCR4	65
	MDA-MB-231 cells	dodecasaccharide	Inhibiting lung metastasis	CXCL12-CXCR4	66
	MDA-MB-231 cell, 4T1 cells	LHTD4	Inhibiting metastasis	CXCL12-CXCR4,TGF-β1	72
	MDA-MB-231 cells	K5-NSOS	Decreasing osteolytic lesion and metastasis tumor burden in bone	TGF-β	74
Pancreatic	MPanc96 cells	S-NACH	Inhibiting adhesion and invasion of cancer cells to endothelial cell	P-selectin	48
Colon	LS180 cells, T84 cells	Heparin	Preventing metastasis	P-selectin	17
	Caco-2 cells	LHTD4/DCK	Inhibiting tumor growth and angiogenesis	N/A	30
	MC-38 mice model	Heparin	Attenuating metastasis lesions	P-selectin	47
	HCT-116 cells	Enoxaparin	Decreased proliferation, adhesion and hepatic metastasis	CXCL12-CXCR4	67
	HT29 cells, HCT-116 cells	G2.2	Inhibiting colonic CSCs	P38 MAP kinase	75
Melanoma	B16-BL6 mouse model	Heparin	Attenuating metastasis lesions	P-selectin	47
	A375 cells, B16F10 cells	RO-heparin, CR-heparin, N-2,3-DS-heparin, 2,3-O-DS-heparin	Inhibiting metastasis	Integrin α_Ⅱb_β_3_	51
	B16F10 cells, MV3 cell	tinzaparin	Inhibiting cancer cells adhesion to endothelium	VLA-4/VCAM-1	53
Lymphoma	Daudi, Ramos, Raji (three kinds of human Burkitt's lymphomas), SU-DHL-6 (human follicular lymphoma), OCI-LY-19 (human Diffused large B-cell lymphoma)	PG545	Eliciting apoptosis	NFκB pathway	37
CSCs	LSCs	CX-01	Promoted chemotherapy efficiency	CXCL12-CXCR4 activity	76
	Hepatoma stem cells	Exogenous heparin	Inhibiting sphere formation	CD44	77
Others	HUVECs, SCC7 cells (murine squamous cell carcinoma)	LHTD4/DCK	Inhibiting tumor growth and anti-angiogenesis	N/A	30
	HUVECs, SCC7 cells	LHsura	Inhibiting proliferation, immigration and endothelial tubular formation	VEGF_165_	32
	HUVECs	PG545	Inhibiting angiogenesis, tumor growth and metastasis	N/A	38

**Table 1.** The abbreviations and their corresponding full names in articles.

Abbreviation	Full name
AML	acute myelogenous leukemia
BMPs	bone morphogenetic proteins
CXCL12	CXC Cytokine Ligand 12
CXCR4	CXC receptor4
CS	chondroitin sulfate
CSC	cancer stem cell
GAG	glycosaminoglycans
ECM	extracellular matrix
ER	endoplasmic reticulum
GPCsR	G protein-coupled receptors
HBD	heparin-binding domain
HDLECs	human dermal lymphatic endothelial cells
HLGAGs	heparin-like glycosaminoglycans
HS	heparan sulfate
HSPG	heparin sulfate proteoglycan protein
HUVECs	human umbilical vein endothelial cells
IL-11	interleukin 11
K5-NSOS	high-molecular-weight Escherichia coli K5-derived heparin-like polysaccharide
LHTD4	low-molecular-weight heparin-taurocholate-tetramer deoxycholate
LHTD4/DCK	a complex of LHTD4 and deoxycholylethylamine
LMWH	Low molecular weight heparin
LHsura	a complex of heparin and suramin fragment
LHbisD4	a conjugation of low molecular weight heparin and four bis-deoxycholates
LSC	leukemic stem cell
PG545	a HS mimetic
PSGL-1	P-selectin glycoprotein ligand-1
S-NACH	Sulfated non-anticoagulant heparin
TCA	taurocholate
TetraDOCA	taurocholate (TCA) and a tetramer of deoxycholic acid
TGFβ/TGFβ1	transforming growth factor/ transforming growth factor beta 1
TGFβ1R1	transforming growth factor beta 1 receptor 1
TKI	tyrosine kinase inhibitor
UFH	unfractionated heparin
VCAM-1	vascular cell adhesion protein-1
VEGF	vascular endothelial growth factor
VEGF-C	vascular endothelial growth factor C
VEGFR-3	vascular endothelial growth factor receptor 3
VLA-4	very late antigen-4

## Heparins act as lymphatic vessel suppressants and angiogenesis inhibitors

Heparin’s biological activity includes inhibition of angiogenesis and lymphogenesis [24], and a number of studies indicate that heparin and its derivatives function as tumor lymphatic vessel and angiogenesis suppressants across a range of cancers. These findings may indicate novel therapeutically-relevant mechanisms by which heparins suppress metastasis. Clinical studies have found that the incidence of cancer metastasis through the lymphatic vessels is 3–5 times higher than through the blood vessels. The vascular endothelial growth factor C (VEGF-C)/vascular endothelial growth factor receptor 3 (VEGFR-3) axis plays an important role in lymphangiogenesis. When VEGFR-3 is phosphorylated by its ligand, a series of downstream signaling pathways trigger lymphangiogenesis, including lymphatic endothelial cell proliferation, migration, and tubular formation [25,26]. Recently, researchers found that LHbisD4, a conjugation made up of LMWH and four bis-deoxycholates, inhibits the formation of new lymphatic vessels by suppressing the phosphorylation of VEGFR-3 induced by VEGF-C [27]. In an *in vitro* study, researchers found that compared with LMWH, in the LHbisD4 treatment group the binding affinity with VEGF-C is significantly higher, but the proliferation, migration and formation of tubular structures are markedly lower. In a 4T1 mouse breast cancer model, LHbisD4 or saline were administrated via an oral route daily for 4 weeks. When the primary 4T1 tumor volume reached 150–200 mm^3^, the lymph node volume indicated that distant metastasis was significantly reduced in the LHbisD4 treatment group compared to the control group. Likewise, LHbisD4 orsaline were administrated via an oral route daily for 8 weeks in an MDA-MB-231 human breast cancer model. The results demonstrated that LHbisD4 stops cancer cells from metastasizing to lymph nodes, so that the volume of lymph nodes does not increase significantly. These findings indicate that heparin and its derivatives are promising candidates for blocking the VEGF-C/VEGFR-3 axis, which can act to reduce lymph node metastasis [28,29].

While there is encouraging evidence of heparin’s ability to inhibit lymphogenesis, other studies have focused on its ability to inhibit angiogenesis. For example, in murine squamous cell carcinoma LHTD4/DCK inhibits tumor growth significantly at a 5 mg/kg dose, with a final tumor volume of 346.9 ± 25.23 mm^3^ in the treatment group, compared with 2561.84 ± 161.65 mm^3^ in the control group [30]. The mechanism underlying this effect appeared to be that LHTD4/DCK inhibited angiogenesis, as the mean blood vessel volume in the LHTD4/DCK group was 8.35 ± 0.4 mm^3^, much lower than 76.19 ± 3.9 mm^3^ in the control group [30]. Yin et al. compared a LMWH and adriamycin combined therapy to adriamycin alone, and found that the combined therapy decreased the lung metastasis of breast cancer cells in C3 H mice, and that heparin inhibited vascular endothelial growth factor (VEGF) expression in tumor tissue and induced cancer cell apoptosis [31]. Another study comparing the effects of LWMH with LHsura found that both inhibit angiogenesis, but the effect of LHsura is much stronger. In a study using human umbilical vein endothelial cells (HUVECs), it was found that LHsura inhibited proliferation, migration and the capillary-like structure formation induced by recombinant VEGF_165_, i.e., simvastatin [32]. The tubular formation inhibitory rate following 50 μg/mL LHsura was 46.4%, compared with 78.6% following 50 μg/mL LMWH. There is a so-called heparin-binding domain (HBD) within the VEGF_165_ molecular structure, which is a 55-residue carboxy-terminal. These enhanced effects were due to the improved affinity of HBD for VEGF_165_ via conjugation with suramin fragments. In addition to inhibiting angiogenesis, heparin and its derivatives can also act to protect the endothelial barrier. For example, the LMWH tinzaparin was found to attenuate VEGF-induced endothelial barrier permeability in a manner that does not depend on its anticoagulant activity [33].

## Heparanase inhibitors as anti-cancer therapeutics

Heparanase is the only enzyme that can lyse heparin sulfate proteoglycan protein (HSPG), by breaking down the extracellular matrix (ECM) and basement membrane. Additionally, heparanase is involved in tumor angiogenesis, invasion and metastasis, and a number of studies suggest that heparanase is a viable target for cancer therapy. As a result, several heparin mimetics have been developed to treat cancer [34–36].

Anticoagulant activity is a common side effect associated with heparin mimetics, but a promising heparin mimetic, PG545 was found to exhibit a strong anti-lymphoma effect and display only mild anticoagulant activity. To study the molecular mechanism underlying the pro-apoptotic effect of PG545, several molecules were measured. The results indicated that PG545 elicits apoptosis via activating the NFκB pathway, inducing endoplasmic reticulum (ER) stress and autophagy [37]. Additionally, PG545 has been found to be a highly potent inhibitor of angiogenesis, tumor growth and metastasis in murine models of breast, liver, lung, prostate, colon, head and neck cancers and melanoma. Sorafenib, a tyrosine kinase inhibitor (TKI), is a well-established drug for treating kidney and liver cancer, but showed no antimetastatic ability in a liver cancer model. However, in combination with sorafenib, PG545 demonstrated enhanced antimetastatic activity and enhanced anti-cancer efficiency in a murine liver cancer model [38].

## Heparin inhibits the metastasis facilitating effect of platelets

It well known that platelets play a key role in the coagulation process, and there is evidence for platelet abnormity amongst cancer patients. Further studies show platelets act as a bridge connecting cancer cells to the endothelial layer, thereby enhancing cancer cell attachment and metastasis [14,22]. Some researchers hold that selectins in platelets trigger the first step of cell-cell interactions, which is relevant to the initiation of tumor metastasis [10,17]. A recent report found that E-cadherin expression in MPanc96-luc cells increased by 2.0 to 2.5-fold after incubation with either S-NACH or tinzaparin [39]. E-cadherin is a marker protein involved in epithelial mesenchymal transition, a key process during malignant tumors’ development of invasion and migration ability. Thus, the above findings suggest the possibility that heparin has its anti-metastasis effect by decreasing the expression of E-cadherin.

**Figure 2.**
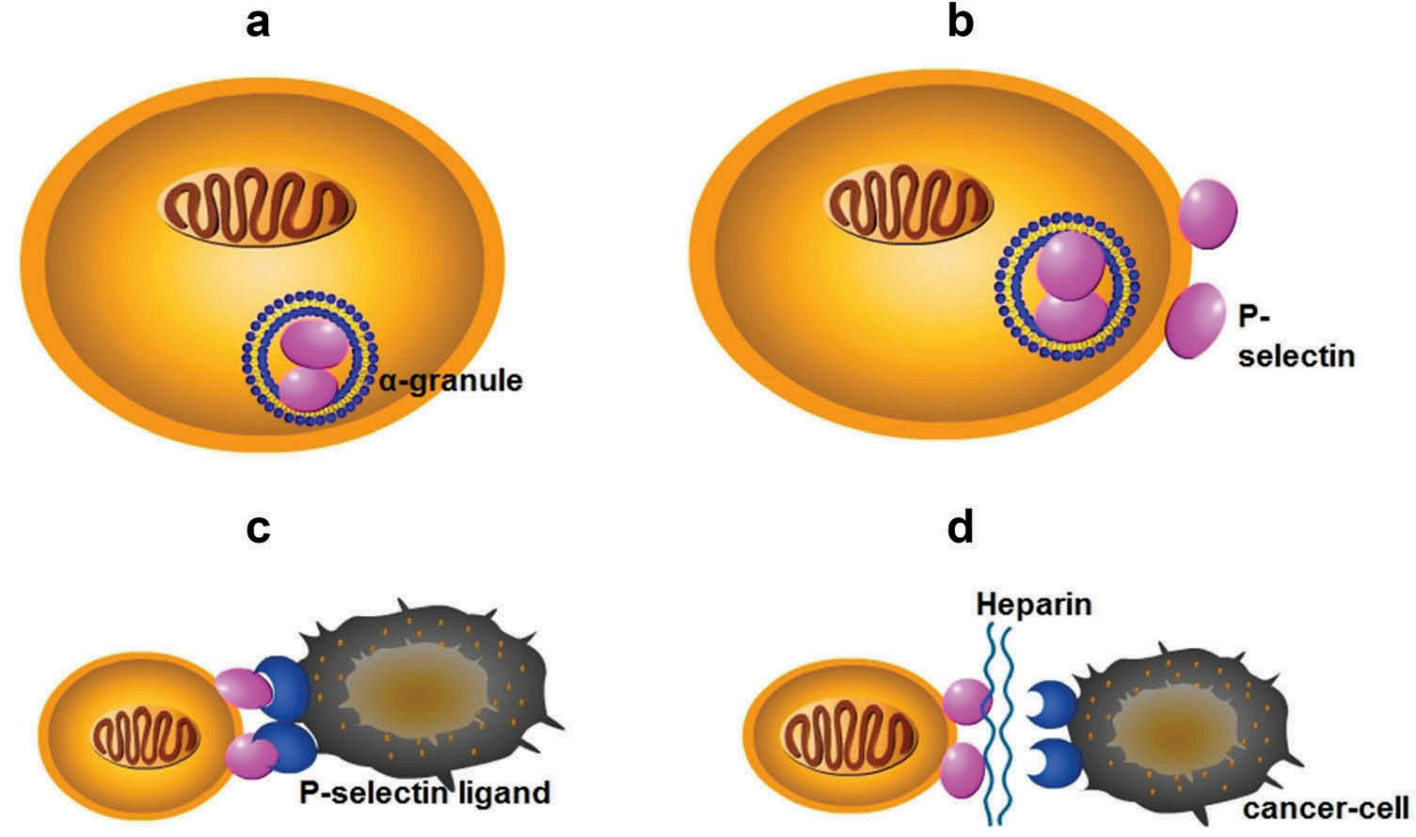
(a): P-selectin is present in the α-granules of platelets; (b): P-selectin in α-granules is rapidly translocated to the cell surface after activation; (c): P-selectin binds to P-selectin ligand on the surface of cancer cells to form a platelet-cancer cell complex, mediating adhesion of cancer cells to endothelial cells; (d): Heparin binds to selectin, blocks the formation of complexes, and interrupts the adhesion of cancer cells.

The selectin family contains three members: P-, E-, and L-selectin. P-selectin is expressed in the storage granules of platelets and endothelial cells, resulting in rapid translocation on cell surfaces upon activation [40]. When P-selectin is absent, the platelet-tumor cell microthrombi degree is minimal, and metastatic lesions in the lungs of mice are subsequently reduced [17,41,42]. L-selectin actively recruits leukocytes and constructs a metastatic niche, while E-selectin is present on activated endothelial cells in the metastatic colonization of the liver [43–46]. Nevertheless, P-selectin has superior effects over L-selectin [10]. Recent research suggests that metastatic lesions are attenuated in modified heparin analogues containing mostly P-selectin inhibitory activity and in P-selectin-deficient mice [47]. In pancreatic cancer, S-NACH dose-dependently inhibits the adhesion and invasion of MPanc96 cancer cells to the endothelial layer of the umbilical cord vein [48]. The adhesion and invasion of Mpanc96 cells are mediated by P-selectin and inhibited effectively by S-NACH. Given these results, heparin binds to P-selectin glycoprotein ligand-1 (PSGL-1) and thereby prevents platelets from binding to cancer cells (Figure 2) [14–17]. Despite the different structures, LMWHs, S-NACH and tinzaparin all target P-selectin to markedly inhibit cancer metastasis in a concentration-dependent manner, which is particularly the case for S-NACH [10,14,18,48]. Furthermore, a greater inhibitory effect was associated with a larger average molecular weight. Additionally, the adhesion between human colon adenocarcinoma LS180 cells and immobilized P-selectin is disrupted by heparin [17]. Taken togehter, results from *in vivo* studies endorse heparin as an efficient inhibitor of selectin-mediated interactions between cancer cells and platelets, providing a possible mechanism for how heparin attenuates cancer metastasis.

**Figure 3.**
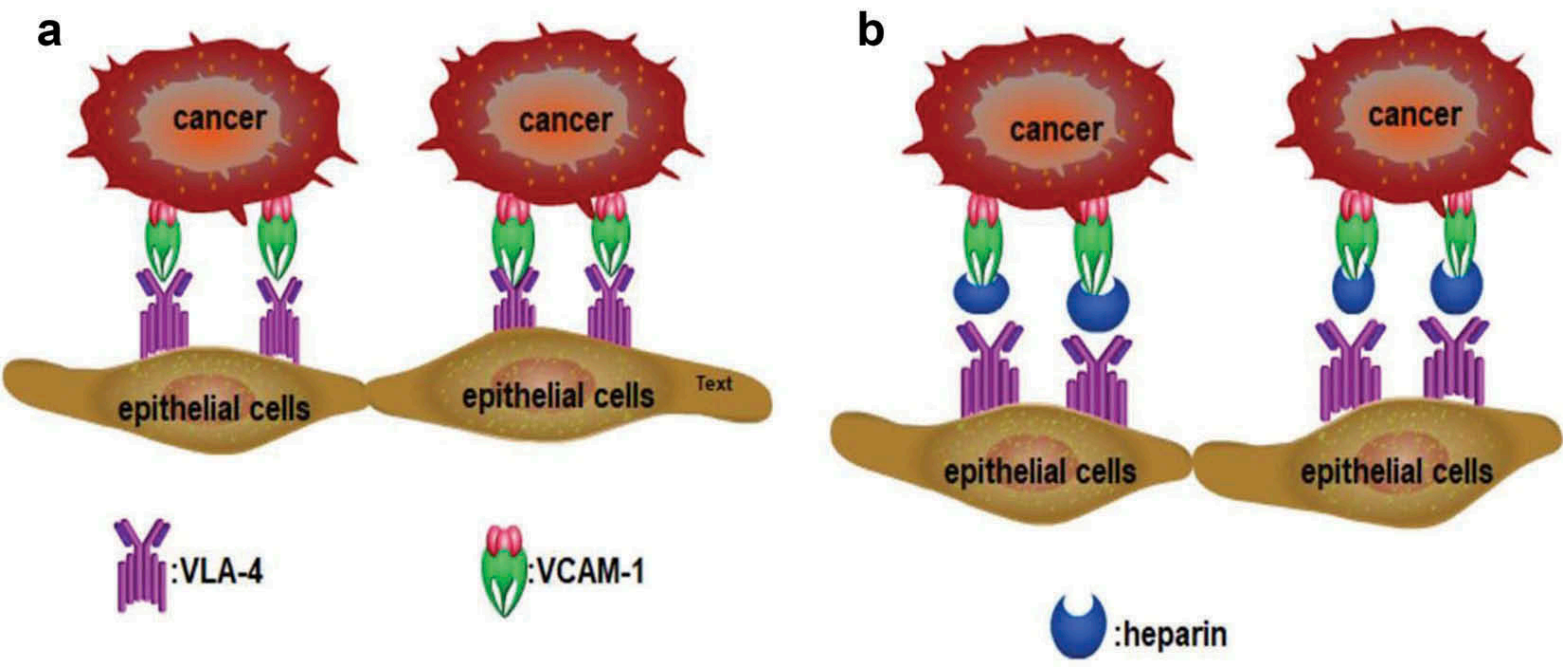
(a): VLA-4 expressed in tumor cells binds to VCAM-1 expressed in endothelial cells, which mediates the adhesion of tumor cells to endothelial cells. (b): Heparin interrupts the binding of VLA-4 to VCAM-1 and inhibits the adhesion of tumor cells to endothelial cells.

Integrins are receptor molecules involved in cell adhesion and signal transmission. Integrin expression by platelets is thought to be a mechanism by which platelets mediate the adhesion of cancer cells to the extracellular matrix, which promotes cancer metastasis. For example, integrin αIIbβ3 is expressed in platelets and is critical in the interaction of platelets with tumor cells [49,50]. Likewise, integrin αMβ2 (Mac-1) mediates the adherence of hematopoietic progenitor cells to the stromal compartment via binding to heparin and heparan sulfate (HS). Moreover, heparin and modified heparin with low anticoagulant activity can inhibit the adhesion of melanoma A375 cells to platelets, which is mediated by integrin αIIbβ3 [51]. Integrin also inhibits the process by which heparin inhibits melanoma cells from adhering to endothelium. Integrin α4β1 (also known as very late antigen-4; VLA-4) binds to vascular cell adhesion protein-1 (VCAM-1) in B16F10 melanoma cells, so that cancer cells adhere to endothelial cells (Figure 3) [52]. Furthermore, heparin can bind to VLA-4 in human melanoma MV3 cells, with binding affinity in the low micromolar range [53]. Structural analysis confirms heparin can bind to integrin, and binding affinity is affected by molecular size, with some short heparin chain or pentasaccharide (Fondaparinux) unable to bind [54]. Binding affinity is also affected by other factors, such as sulfation density [55].

## Heparin and cytokines

Cytokines are small soluble proteins produced by cells that are involved in oncogenesis and the development of cancer. The possible influence of heparin and its derivatives on cytokines has been a focus in understanding how heparin affects cancer cells and cancer progression. Among the different types of cytokines, chemokines are low molecular weight proteins that induce white blood cell migration, which plays an important role in inflammation. They are small, secreted proteins that induce cell migration through activation of G protein-coupled receptors (GPCsR), and bind to extracellular matrix GAG in order to direct chemotaxis along a gradient of increasing chemokine concentration. A substantial number of studies highlight the involvement of chemokines and their receptors in cancer metastasis. The presentation of chemokines to their receptors relies on GAG components on the cell surface, and GAG-binding is essential for the cell migration stimulated by chemokines [56]. Additional evidence suggests the activity of chemokines is directly regulated by GAGs [57].

**Figure 4.**
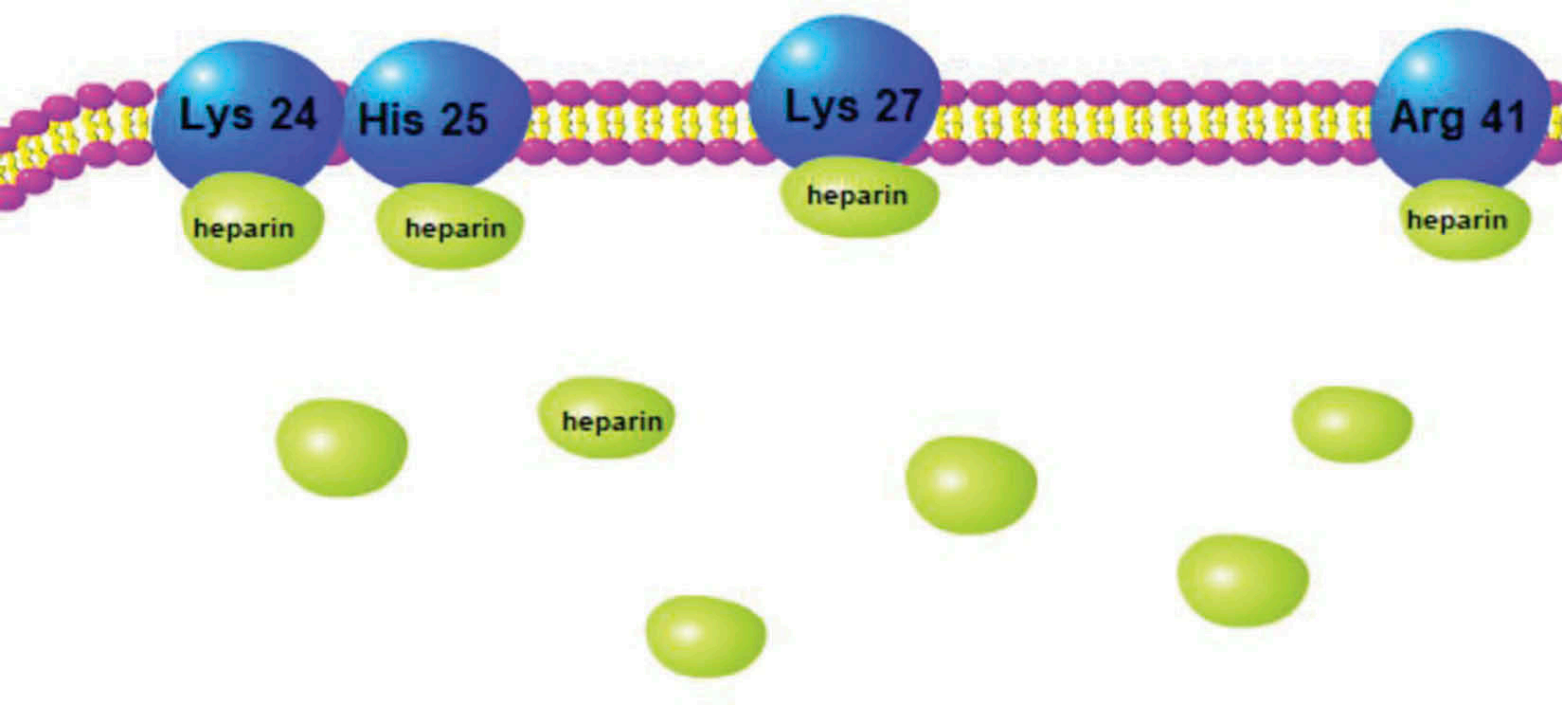
Binding site of heparin to CXCL12 dimer.

**Figure 5.**
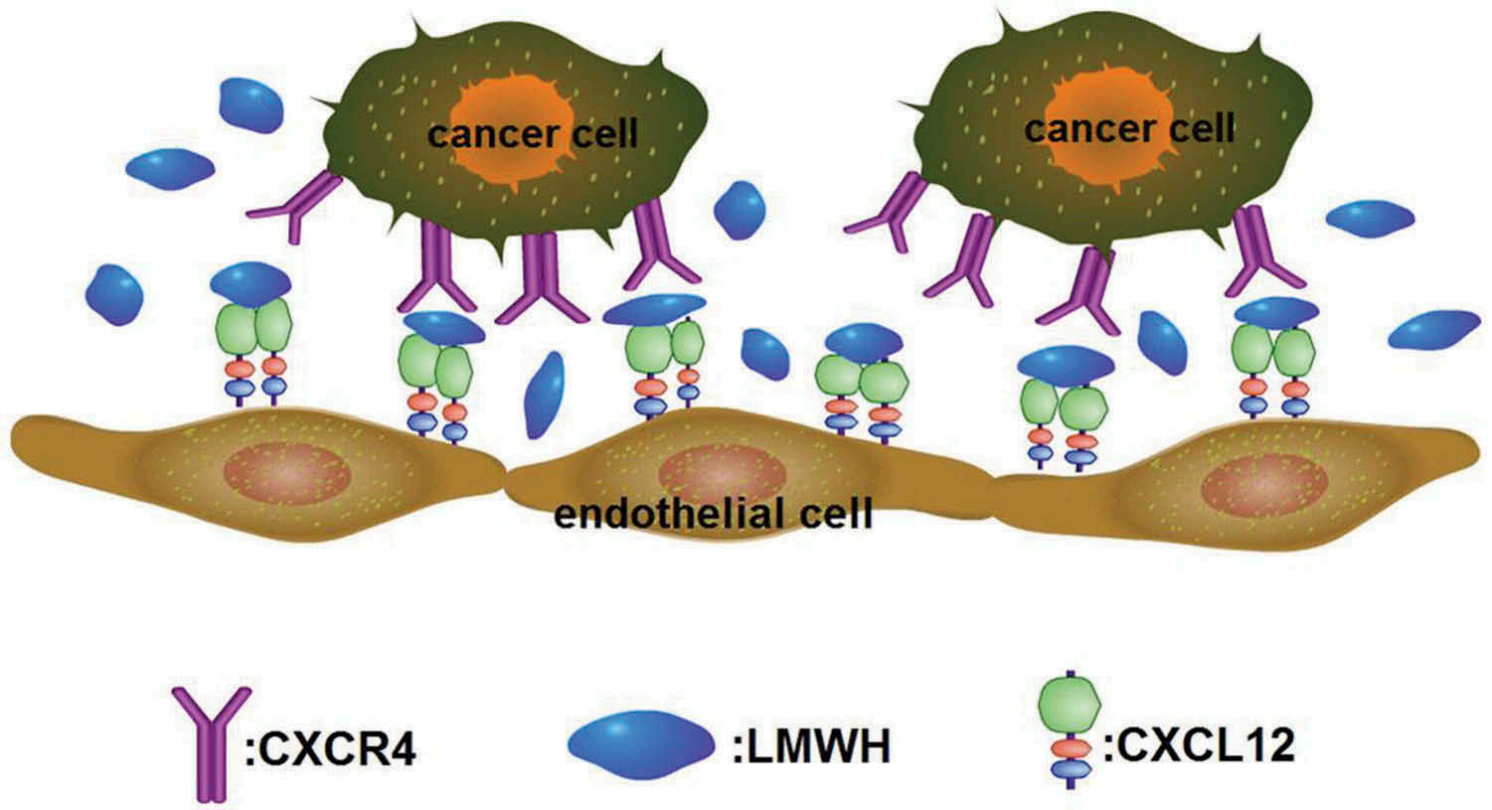
CXCR4 on tumor cells surface binds to its ligand CXCL12 expressed in endothelial cell membrane to promote the metastasis of tumor cells. LMWH binds to CXCL12 to make it a dimerization state, blocking its binding to CXCR4 and inhibiting metastasis.

The arrangement of conserved cysteine residues near the amino terminus indicates that chemokines consist of four families, C, CC, CXC and CX3 C. Among them, CXC Cytokine Ligand 12 (CXCL12, formerly known as stromal cell-derived factor-1, SDF1) and CXC receptor 4 (CXCR4) comprise the CXCL12-CXCR4 axis, which is widely acknowledged as playing a vital role in cancer metastasis. The CXCL12-CXCR4 axis promotes cancer development mainly through two mechanisms: 1) CXCR4-expressing cells are located in CXCL12-expressing organs; and 2) Elevated CXCL12 levels regulate the survival, growth, metastasis and angiogenesis of cancer cells via paracrine signaling [58,59]. In fact, the CXCL12/CXCR4 axis is involved in various physiological functions, such as the entrance of neutrophils into infection sites, stem cell mobilization and directed migration [60–63]. Recent research indicates that heparin’s anti-metastatic ability may be underpinned by the modulation of the CXCR4-CXCL12 axis [64]. For example, heparin and Tinzaparin reduced the pulmonary metastasis of breast cancer cells that were over expressing CXCR4 by interfering with the interaction of CXCL12 and its receptor CXCR4 [65,66]. In a study of human colon cancer cells HCT-116, their proliferation, adhesion and colony formation were promoted by CXCL12, and this process was inhibited by enoxaparin. Additionally, CXCR4 expression in hepatic sinusoidal endothelial cells is down-regulated along with the significant decrease of hepatic metastasis after enoxaparin treatment [67]. Other evidence suggests that CXCR4 mediates the interactions between cancer cells and stroma cells by combining with its natural ligand CXCL12 [68,69]. The CXCL12-CXCR4 axis also mediates the migration of breast cancer cells and their seeding in distant organ tissues, but heparin blocks this interaction; the specific binding sites are shown in Figure 4 [70]. LMWH binds to a heparin-binding site in CXCL12, making CXCL12 a dimerization shift from the monomer dimer equilibrium (Figure 5), and LMWH consequently decreases CXCR4-CXCL12 interaction [71].

In a study that developed a transplant tumor model of 4T1 breast cancer in mice, treatment with 5 mg/kg/daily LHTD4, taurocholate (TCA) and a tetramer of deoxycholic acid (tetraDOCA) for 8 weeks significantly reduced the formation of metastases [72]. LHTD4 inhibited the migration of MDA-MB-231 cancer cells by blocking the transforming growth factor beta 1 (TGF-β1) signaling pathway and the CXCL12-CXCR4 axis. Specifically, LHTD4 inhibits TGFβ1-mediated phosphorylation of TGF-TGFβ1R1 as well as TGFβ1-induced vimentin and SNAIL-1 expression. Meanwhile, LHTD4 blocks CXCL12-induced CXCR4 phosphorylation and subsequent ligand-receptor response, cell migration and invasion [72].

Heparin promotes bone resorption by enhancing the activity of osteoclasts and inhibits bone formation by weakening the function of osteoblasts. During osteoclastic bone resorption, first TGFβ and then osteolytic factors (e.g. interleukin 11; IL-11) are released. TGFβ regulates several steps in cancer metastasis, including the establishment of bone metastatic lesions. Specifically, UFH is more effective than LMWH at stimulating osteoclast and inhibiting osteoblast activity [73]. K5-NSOS can inhibit TGFβ–induced IL-11, and effectively decrease the osteolytic lesion area and metastatic tumor burden in bones, but markedly alleviates the body weight loss and tumor-related cachexia in a breast cancer bone metastasis mouse model [74].

## Heparin prevents cancer relapse by inhibiting cancer stem cells

Inhibiting cancer stem cells (CSCs) is a critical mechanism being proposed for preventing cancer relapse and targeting CSCs is expected to be a promising approach for cancer treatment. Various mechanisms are under consideration in identifying how heparin or heparin-like molecules modulate CSCs. For instance, G2.2, a sulfated nonsaccharide GAG mimetic of heparin hexasaccharide was found to selectively inhibit colonic CSCs *in vitro, in vivo* and *ex vivo*. The CSC self-renewal inhibiting function of G2.2 was mediated through p38 MAP kinase activation [75].

As shown in Figure 5, heparin can inhibit cancer metastasis by blocking the binding of CXCL12 and CXCR4. CXCL12/CXCR4 also mediates the sequestration of the quiescent leukemic stem cells (LSCs) in marrow. These LSCs will cause chemotherapy resistance in acute myelogenous leukemia (AML). CX-01 is a low-anticoagulant heparin derivative that can block CXCL12/CXCR4 activity and therefore disrupt the LSCs in marrow, and CX-01 has been clinically verified to promote chemotherapy efficiency in the treatment of AML [76]. In addition to affecting AML, the same mechanisms support the therapeutic potential of CX-01 for myelodysplastic syndrome, multiple myeloma, and lymphoma.

GAGs HS and chondroitin sulfate (CS) have been reported to regulate self-renewal and pluripotency of CSCs thorough cellular signaling. Recent evidence demonstrates that exogenous CS enhanced hepatoma sphere formation by blocking specific protein binding to CD44, while the addition of exogenous heparin inhibited sphere formation, indicating that heparin and its derivatives are potential candidates for reducing hepatoma stem cells [77].

Other studies focused on the safety of heparin and its derivatives. M. Reza Sadaie [78] demonstrated that heparin and its derivatives could influence the release and expansion of bone CSCs. Bone morphogenetic proteins (BMPs) either potentiate or inhibit the growth of bone CSCs. Heparin and its derivatives bind to BMPs and modulate CSCs positively or negatively, which subsequently has either positive or negative effects on tumorigenesis.

## Conclusions and prospects

Heparin is used in the prevention and treatment of venous thromboembolism for cancer patients owing to its strong anticoagulant activity. Clinical studies suggest anticoagulant therapy with heparin leads to better prognosis and survival for patients with diversiform tumors. These findings have revealed that heparin is not only an effective anticoagulant, but may also be an undiscovered novel anti-cancer agent. A growing body of evidence suggests that heparin can decelerate the development of cancer and metastases. However, for both UFH and LMWH, the risk of bleeding caused by anticoagulants limits the application of traditional heparin in cancer treatment, leading to the development of a number of synthetic heparin derivatives. Compared with traditional heparin, these new synthetic heparin derivatives possess much lower anticoagulant activity, but maintain the basic structure and biological functions of heparin. Moreover, the conjugated group also increases the affinity of heparin to its target molecule. As outlined in this article, various types of heparin and its derivatives can inhibit tumor cell proliferation, migration, invasion, and enhance the chemosensitivity of tumor cells. Heparin derivatives modulate hematogenous metastasis by inhibiting the interaction between platelets and tumor cells, and control lymphatic metastasis by inhibiting lymphangiogenesis. Furthermore, heparin derivatives bind to a range of heparin-binding proteins to block signal pathways including TGF-β1, integrins, CXCL12-CXCR4 axis, and VEGF-C/VEGFR-3 axis. Moreover, several mimetics of heparin have been developed as anti-cancer agents. For instance, G2.2 was found to selectively inhibit colonic CSCs in vivo [75]. Due to their potential anti-metastatic ability and good biocompatibility, heparin and its derivatives have been used in the construction process in nanomedicine, with results indicating that nanoparticles based on heparin and its derivatives are promising agents for postoperative chemotherapy [79]. Heparin and its derivatives such as LMWH and S-NACH can enhance the uptake of chemotherapeutics, as demonstrated in an *in vivo* study with a xenograft cancer model [80]. Consequently, heparin and its derivatives should be developed as promising new adjuvant anti-cancer drugs which can operate via a range of pathways to affect multiple stages of tumor progression.
